# Häufigkeit und Charakteristika von Einsätzen der Gemeindenotfallsanitäter bei Pflegebedürftigen: Analyse von 2410 Einsatzprotokollen bei Personen im Alter von 65+ Jahren

**DOI:** 10.1007/s00063-023-01085-w

**Published:** 2023-12-06

**Authors:** Andrea Diana Klausen, Ulf Günther, Guido Schmiemann, Falk Hoffmann, Insa Seeger

**Affiliations:** 1 Fakultät VI Medizin und Gesundheitswissenschaften, Carl von Ossietzky Universität Oldenburg, Ammerländer Heerstr. 140, 26129 Oldenburg, Deutschland; 2 Department für Versorgungsforschung, Carl von Ossietzky Universität Oldenburg, Oldenburg, Deutschland; 3 Klinikum Oldenburg AöR, Universitätsklinik für Anästhesiologie | Intensivmedizin | Notfallmedizin | Schmerztherapie, Oldenburg, Deutschland; 4 Abteilung Versorgungsforschung, Institut für Public Health und Pflegeforschung (IPP), Universität Bremen, Bremen, Deutschland; 5 Health Sciences Bremen, Universität Bremen, Bremen, Deutschland

**Keywords:** Notfallversorgung, Pflegebedürftigkeit, Primärversorgung, Rettungsdienst, Versorgungsforschung, Emergency medicine, Emergency medical services, Health services research, Long-term care, Primary care

## Abstract

**Hintergrund:**

Ziel der Arbeit war eine differenzierte Betrachtung der Rettungsdiensteinsätze von Gemeindenotfallsanitätern (G-NFS) bei älteren Pflegebedürftigen im Pflegeheim und in der Häuslichkeit.

**Methodik:**

Retrospektive Auswertung aller G‑NFS-Einsatzprotokolle aus dem Jahr 2021 von älteren Patienten (≥65 Jahre), unterteilt nach den Einsatzorten Pflegeheim, häusliche Pflege und nichtpflegebedürftig. Es wurden Maßnahmen, Dringlichkeit, Transport und Empfehlung deskriptiv analysiert.

**Ergebnisse:**

Von 5900 G‑NFS-Protokollen entfielen 43,0 % (*n* = 2410) auf ältere Patienten (Durchschnittsalter 80,8 Jahre, 49,7 % weiblich). Die Einsätze erfolgten mit 20,6 % (*n* = 496) bei Pflegeheimbewohnern, 38,4 % (*n* = 926) bei Pflegebedürftigen in häuslicher Versorgung und 41 % (*n* = 988) bei Nichtpflegebedürftigen. Eine Beratung erhielten 48,4 % der Pflegeheimbewohner, 82,1 % der Pflegebedürftigen in häuslicher Versorgung und 83,7 % der Nichtpflegebedürftigen. Etwa 60 % der Einsätze wurden bei allen Einsatzorten als nichtdringlich eingestuft. Auf Transporte wurde bei 63,1 % der Pflegeheimbewohner, 58,1 % der Pflegebedürftigen in häuslicher Versorgung und 60,6 % der Nichtpflegebedürftigen verzichtet. Ein Besuch der Notaufnahme wurde 29,4 % der Pflegeheimbewohner, 37,6 % der Pflegebedürftigen in häuslicher Versorgung und 33,6 % der Nichtpflegebedürftigen empfohlen. Dauerkatheter wurden häufiger im Pflegeheim (38,5 %) versorgt als in häuslicher Versorgung (15,1 %) und bei Nichtpflegebedürftigen (9,3 %).

**Schlussfolgerungen:**

G‑NFS übernehmen primärversorgende Aufgaben und können zu einer Reduzierung unnötiger Transporte beitragen. Es muss jedoch diskutiert werden, inwieweit der Rettungsdienst für solche Einsätze zuständig ist und wie ältere Pflegebedürftige zukünftig bedarfsgerecht versorgt werden können.

**Zusatzmaterial online:**

Zusätzliche Informationen sind in der Online-Version dieses Artikels (10.1007/s00063-023-01085-w) enthalten.

## Abstract (en)

**Background:**

The aim of this study was to obtain a differentiated view of interventions delivered by community paramedics (*Gemeindenotfallsanitäter*, G‑NFS) in older people in need of care living in nursing homes and at home.

**Materials and methods:**

A retrospective analysis of G‑NFS documentation from 2021 with a focus on patients aged ≥65 years was performed. Data were grouped into callouts to nursing homes or private homes. Interventions, urgency, transport, and further recommendations were analyzed descriptively.

**Results:**

Of 5,900 G-NFS protocols, 43.0% (*n* = 2,410) were related to elderly people (mean age 80.8 years, 49.7% female). A total of 20.6% of these callouts involved nursing home residents, 38.4% (*n* = 926) were homecare patients, and 41% (*n* = 988) of callouts were to persons who did not rely on care. No specific interventions except advice were given to 48.4% of nursing home residents, and to even 82.1% of those in homecare and 83.7% of those without care needs. About 60% of the G‑NFS interventions were classified as non-urgent. Transport was waived for 63.1% of nursing home residents, for 58.1% in homecare, and for 60.6% of persons without care needs. A visit to the emergency department was recommended to 29.4% of nursing home residents, 37.6% of homecare patients, and 33.6% of persons without need of care. Measures related to urine catheters were documented much more often in nursing homes (38.5%) than in patients in homecare (15.1%) or without need of care (9.3%).

**Conclusion:**

Community paramedics perform primary care tasks and can contribute to a reduction in unnecessary transport. It should be discussed whether the emergency medical service is responsible for such interventions and how older people in need of care can be cared for according to their needs in the future.

Der Anteil pflegebedürftiger und geriatrischer Patienten steigt. Anders als jüngere Patienten hat diese Klientel einen erhöhten Versorgungsbedarf, der mit einer höheren Inanspruchnahme von Versorgungsleistungen, zum Beispiel Rettungsdiensteinsätze, einhergeht [1, 20]. Ältere Menschen ab dem 65. Lebensjahr machen etwa 40 % aller Notaufnahmepatienten aus und werden häufiger rettungsdienstbegleitet als Jüngere [14]. Mit zunehmendem Alter steigt auch der Anteil Pflegebedürftiger, diese Personen werden entweder von formell bzw. informell Pflegenden in der Häuslichkeit oder in Pflegeheimen versorgt [12]. Im Setting Pflegeheim erfolgt häufig eine Versorgung durch den Rettungsdienst [5, 13]. In Pflegeheimen und in der Häuslichkeit nimmt ferner der Anteil der Patienten zu, die aus haftungsrechtlichen Gründen in Notaufnahmen transportiert werden [16]. Nicht alle diese Patienten haben vital bedrohliche Erkrankungen, die einer Notfallversorgung im Krankenhaus bedürfen [21].

Mit dem Projekt „Gemeindenotfallsanitäter“ (G-NFS) wurde im Oldenburger Land im Jahr 2019 von vier Rettungsdienstträgerschaften ein Pilotprojekt initiiert, das Notfallsanitäter speziell qualifiziert [6]. Eine erste Studie aus dem Jahr 2019/2020 zeigte, dass 14 % aller G‑NFS-Einsätze bei älteren Menschen in Pflegeheimen stattfanden [18]. Die vorhandenen Leistungsdaten dieser Jahre ermöglichten jedoch keine Unterscheidung, ob auch bei Einsätzen in der Häuslichkeit Pflegebedürftige versorgt wurden. Zu Rettungsdiensteinsätzen bei Pflegebedürftigen in häuslicher Versorgung in Deutschland liegt bisher insgesamt kaum Literatur vor. Um mögliche Versorgungslücken aufzudecken und die von den G‑NFS erbrachten Leistungen besser darstellen zu können, erfolgte deshalb eine Anpassung der G‑NFS-Einsatzprotokolle.

Ziel der vorliegenden Arbeit ist eine differenzierte Darstellung der Einsatzcharakteristika der G‑NFS bei Pflegebedürftigen im Setting Pflegeheim und in der Häuslichkeit sowie der Vergleich zu Nichtpflegebedürftigen.

## Methodik

### Datenbasis und Studiendesign

Datenbasis dieser retrospektiven Beobachtungsstudie bilden die G‑NFS-Einsatzprotokolle für den Zeitraum 01.01.–31.12.2021, die zusätzlich zum Einsatzprotokoll der Deutschen Interdisziplinären Vereinigung für Intensiv- und Notfallmedizin (DIVI) von den G‑NFS im Rahmen der Leistungserbringung ausgefüllt und als Sekundärdaten anonymisiert zur Verfügung gestellt wurden. In die Auswertung wurden alle G‑NFS-Einsatzprotokolle von Patienten eingeschlossen, die 65 Jahre und älter waren. Das Vorhaben wurde von der Medizinischen Ethikkommission der Universität Oldenburg positiv bewertet (Votums-Nr. 2019-30).

### Setting und Implementierung des G-NFS

Seit dem 01.01.2019 werden in den vier niedersächsischen Kommunen Stadt Oldenburg sowie die Landkreise Ammerland, Cloppenburg und Vechta mit insgesamt rund 615.600 Einwohnern im Rahmen eines Pilotprojekts Notfallsanitäter zu G‑NFS ausgebildet [6]. Das Rettungsmittel G‑NFS wird 24/7 eingesetzt. Eine Alarmierung der G‑NFS erfolgt durch die zuständigen Leitstellen und umfasst niedrigprioritäre Rettungsdiensteinsätze, für die nach standardisierter und strukturierter Notrufabfrage keine ärztliche Intervention oder ein Transport als notwendig erachtet wird. Ein G‑NFS besetzt ausschließlich allein das Einsatzfahrzeug. Die Fahrt zum Einsatzort erfolgt ohne Sonderrechte, mit Ausnahme der Alarmierung als First Responder. Nach erstem Patientenkontakt führen die G‑NFS eine Anamnese und ggf. eine körperliche Untersuchung durch. Vor Ort werden erste pflegerisch-medizinische Maßnahmen durchgeführt oder Entscheidungen für eine weitere medizinische Versorgung getroffen. Bei Bedarf besteht die Möglichkeit, ärztliche Expertise durch die Telemedizinzentrale des Klinikums Oldenburg zu erhalten oder zusätzliche Rettungsmittel über die Leitstellen nachzufordern.

### Einsatzprotokolle und erhobene Daten

Die G‑NFS dokumentieren nach jedem Einsatz Basischarakteristika zur Versorgung und zu den Patienten. Es werden Daten zur Wohnsituation (Pflegeheim/Häuslichkeit), die Pflegesituation in der Häuslichkeit (häusliche Pflege/nicht pflegebedürftig), zur Behandlungsdringlichkeit gemäß Patientenzuweisungscode (PZC) [8] sowie demografische Daten erhoben (Geschlecht, Geburtsjahr). Weiterhin erfassen die G‑NFS Angaben zu folgenden Kategorien: durchgeführte Maßnahmen, Inanspruchnahme Telemedizin, Notwendigkeit eines Transports, Empfehlungen zur weiteren Versorgung, vorherige Kontaktaufnahme zum Hausarzt/Bereitschaftsdienst durch die Patienten, Kontaktaufnahme zum Hausarzt/Bereitschaftsdienst durch G‑NFS, Einschätzung des G‑NFS zur Kategorisierung des Einsatzes durch die Leitstelle (Zusatzmaterial online: S1 Methodik). Die Angaben im Protokoll waren als Einfach- oder Mehrfachantwort zu beantworten.

Nach Übermittlung der Protokolle an die Universität Oldenburg wurden diese mithilfe der Software TeleForm (Electric Paper Informationssysteme, Lüneburg, Deutschland, Version 16.5) eingelesen und als Comma-separated values Datei (CSV-Datei) exportiert. Von der Erfassungssoftware fehlerhaft oder inkorrekt abgebildete Wiedergaben der Auswahl- oder Freitextfelder wurden manuell korrigiert.

### Statistische Analyse

Die Auswertung der Daten erfolgte deskriptiv. Alle Analysen wurden unterteilt nach Pflegebedarf und Versorgungssetting (Pflegeheim, häusliche Pflege und nicht pflegebedürftig). Ausgewertet wurden die durchgeführten Maßnahmen, die Konsultation von Hausarzt/KV-Notdienst, die Nutzung von Telemedizin, Empfehlungen zur weiteren Versorgung und ob ein Transport erfolgte. In der Kategorie Maßnahmen wurden Antwortmöglichkeiten, die einer Medikationsgabe zuzuordnen sind, zusammengefasst. Dies traf auch auf Antwortmöglichkeiten, die der Versorgung transurethraler und suprapubischer Urindauerkatheter zuzuordnen waren („Entfernung Dauerkatheter“ und „Wiederanlage Dauerkatheter“ sowie entsprechende Freitextangaben) zu (Zusatzmaterial online: S2 Maßnahmen). Weiterhin wurden zusätzlichen Kategorien für Alter (65–74, 75–84, 85+ Jahre) und Einsatzdauer (weniger als 30, 30–59, 60–89 und 90+ min) gebildet und ausgewertet.

Die Auswertung erfolgte mittels der Statistiksoftware IBM SPSS Statistics for Windows (Version 27.0. SPSS Inc., Chicago/IL, USA) und SAS für Windows Version 9.4 (SAS Institute Inc., Cary/NC, USA).

## Ergebnisse

### Baseline-Charakteristika

Im Erhebungszeitraum wurden insgesamt 5900 G-NFS-Protokolle vollständig ausgefüllt, davon entfielen 2410 (43,0 %) auf Patienten im Alter von 65+ Jahren. Diese waren durchschnittlich 80,8 Jahre alt, und 49,7 % (*n* = 1176) waren weiblich. Mehr als die Hälfte der Einsätze (*n* = 1354, 59,3 %,) wurde von den G‑NFS retrospektiv als nicht dringlich eingestuft (PZC = 0). Die Einsatzdauer betrug durchschnittlich 45 min (Tab. 1).

**Table 1:** 

	Gesamt 100 % (*n* = 2410)	Pflegeheim 20,6 % (*n* = 496)	Pflegebedürftige in häuslicher Versorgung 38,4 % (*n* = 926)	Nicht pflegebedürftig 41,0 % (*n* = 988)
Alter in Jahren, Mittelwert (SD)	80,8 (8,2)	83,3 (7,9)	82,6 (7,8)	77,8 (7,7)
*Altersgruppen (n* *=* *2410)*				
65–74 Jahre	25,5 % (*n* = 614)	16,9 % (*n* = 84)	17,5 % (*n* = 162)	37,2 % (*n* = 368)
75–84 Jahre	41,5 % (*n* = 1000)	35,3 % (*n* = 175)	41,8 % (*n* = 387)	44,3 % (*n* = 438)
85+ Jahre	33,0 % (*n* = 796)	47,8 % (*n* = 237)	40,7 % (*n* = 377)	18,4 % (*n* = 182)
*Geschlecht (n* *=* *2367)*				
Männer	50,3 % (*n* = 1191)	62,1 % (*n* = 183)	48,9 % (*n* = 465)	45,8 % (*n* = 528)
Frauen	49,7 % (*n* = 1176)	37,9 % (*n* = 300)	51,1 % (*n* = 445)	54,2 % (*n* = 446)
*First Responder (n* *=* *2410)*	4,7 % (*n* = 114)	8,1 % (*n* = 40)	3,1 % (*n* = 29)	4,6 % (*n* = 45)
*Dringlichkeit des Einsatzes (n* *=* *2285)*				
PZC 0 (nicht dringlich)	59,3 % (*n* = 1354)	59,5 % (*n* = 273)	57,5 % (*n* = 508)	60,8 % (*n* = 573)
PZC 1 (sofortiger Arztkontakt)	4,6 % (*n* = 105)	4,4 % (*n* = 20)	4,1 % (*n* = 36)	5,2 % (*n* = 49)
PZC 2 (stationäre Aufnahme wahrscheinlich, kein unmittelbarer Handlungsbedarf)	20,8 % (*n* = 476)	19,4 % (*n* = 89)	24,2 % (*n* = 214)	18,4 % (*n* = 173)
PZC 3 (vermutlich ambulante Behandlung ausreichend)	15,3 % (*n* = 350)	16,8 % (*n* = 77)	14,3 % (*n* = 126)	15,6 % (*n* = 147)
*Einsatzdauer in Minuten, Mittelwert (SD)*	45,8 (21,0)	43,1 (21,0)	45,5 (21,9)	47,4 (19,9)
*Einsatzdauer in Gruppen (n* *=* *2378)*				
< 30 min	21,0 % (*n* = 499)	27,0 % (*n* = 132)	22,6 % (*n* = 207)	16,4 % (*n* = 160)
30–59 min	56,3 % (*n* = 1340)	54,3 % (*n* = 264)	55,4 % (*n* = 506)	58,3 % (*n* = 569)
60–89 min	18,2 % (*n* = 432)	14,5 % (*n* = 71)	17,0 % (*n* = 155)	21,1 % (*n* = 206)
90+ min	4,5 % (*n* = 107)	4,1 % (*n* = 20)	5,0 % (*n* = 46)	4,2 % (*n* = 41)

Von den 2410 Einsätzen entfielen 20,6 % (*n* = 496) der Einsätze auf Pflegeheimbewohner, 38,4 % (*n* = 926) auf Pflegebedürftige in häuslicher Versorgung und 41 % (*n* = 988) auf Nichtpflegebedürftige (Tab. 1). Das Durchschnittsalter der Nichtpflegebedürftigen lag mit 77,8 Jahren unter dem der Pflegeheimbewohner (83,3 Jahre) und dem der Pflegebedürftigen in häuslicher Versorgung (82,6 Jahre). Der Anteil männlicher Patienten war im Pflegeheim am höchsten. Es gab keine relevanten Unterschiede in Bezug auf Einsatzzeiten und Dringlichkeit der Versorgung (Tab. 1).

### Maßnahmen der G-NFS

Bei den 2410 Einsätzen bei Pat. im Alter von 65+ Jahren wurden am häufigsten die Maßnahmen Beratung (*n* = 1827; 75,8 %), Medikationsgabe (*n* = 438; 18,2 %), Hilfe zur Selbstmedikation (*n* = 406; 16,8 %) und Versorgung von Dauerkathetern (*n* = 365; 15,1 %) dokumentiert (Supplement 2).

Eine Beratung durch G‑NFS erhielten fast die Hälfte der Pflegeheimbewohner (*n* = 240; 48,4 %) sowie acht von 10 Pflegebedürftigen in häuslicher Versorgung (*n* = 760; 82,1 %) und Nichtpflegebedürftige (*n* = 827; 83,7 %). Eine Versorgung von Dauerkathetern wurde bei mehr als einem Drittel der Einsätze im Pflegeheim (*n* = 191; 38,5 %) dokumentiert, deutlich seltener bei pflegebedürftigen Patienten in häuslicher Versorgung (*n* = 140; 15,1 %) und Nichtpflegebedürftigen (*n* = 34; 3,4 %). Die Maßnahme Medikationsgabe wurde bei Pflegeheimbewohnern (*n* = 72; 14,5 %) und Pflegebedürftigen in häuslicher Versorgung (*n* = 147; 15,9 %) seltener durchgeführt als bei Nichtpflegebedürftigen (*n* = 219; 22,2 %). Hilfe bei Selbstmedikation erhielten Pflegeheimbewohner (*n* = 46; 9,3 %) ebenfalls seltener als Pflegebedürftige in häuslicher Versorgung (*n* = 169; 18,3 %) und Nichtpflegebedürftige (*n* = 191; 19,3 %). Insgesamt erfolgte bei einem höheren Anteil der männlichen Patienten die Versorgung eines Dauerkatheters (*n* = 302; 25,4 % vs. *n* = 54; 4,6 %). Dies spiegelte sich in allen Settings wider. Bei Frauen wurden häufiger Beratungen und Medikationsgaben dokumentiert.

### Weiterversorgung und Empfehlungen der Gemeindenotfallsanitäter

In 6,4 % (*n* = 154) aller Einsätze wurde ein Hausarzt oder der KV-Notdienst durch den G‑NFS hinzugezogen. Eine telemedizinische Konsultation erfolgte bei weniger als einem Prozent der Einsätze (*n* = 19; 0,8 %) (Tab. 2). Nach Erstversorgung wurde einem Drittel der versorgten Patienten eine Vorstellung in der Notaufnahme (*n* = 826; 34,3 %) bzw. beim Hausarzt (*n* = 891; 37,0 %) empfohlen. Eine ambulante Versorgung vor Ort ohne Transport wurde bei 60,2 % (*n* = 1389) aller Einsätze durchgeführt.

**Table 2:** 

	Gesamt in % (*n* = 2410)	Pflegeheim in % (*n* = 496)	Pflegebedürftige in häuslicher Versorgung in % (*n* = 926)	Nicht pflegebedürftig in % (*n* = 988)
Nutzung von Telemedizin	0,8 (*n* = 19)	1,4 (*n* = 7)	0,3 (*n* = 3)	0,9 (*n* = 9)
Konsultation Hausarzt/KV-Notdienst	6,4 (*n* = 154)	6,3 (*n* = 31)	6,2 (*n* = 57)	6,7 (*n* = 66)
*Empfehlung weitere Versorgung*				
Vorstellung Notaufnahme	34,3 (*n* = 826)	29,4 (*n* = 146)	37,6 (*n* = 348)	33,6 (*n* = 332)
Vorstellung beim Hausarzt	37,0 (*n* = 891)	23,6 (*n* = 117)	36,6 (*n* = 339)	44,0 (*n* = 435)
Vorstellung beim Facharzt	7,6 (*n* = 183)	13,5 (*n* = 67)	8,1 (*n* = 75)	4,1 (*n* = 41)
Vorstellung KV-Notdienst	4,2 (*n* = 101)	2,4 (*n* = 12)	2,8 (*n* = 26)	6,4 (*n* = 63)
*Transport (n* *=* *2309)*				
KTW (Krankentransportwagen)	13,0 (*n* = 301)	15,8 (*n* = 73)	16,4 (*n* = 148)	8,5 (*n* = 80)
N‑KTW (Notfallkrankenwagen)	11,2 (*n* = 259)	10,4 (*n* = 48)	12,6 (*n* = 114)	10,3 (*n* = 97)
RTW (Rettungswagen)	10,1 (*n* = 234)	10,2 (*n* = 47)	8,0 (*n* = 72)	12,2 (*n* = 115)
Nachforderung Notarzteinsatzfahrzeug	1,6 (*n* = 37)	0,7 (*n* = 3)	0,9 (*n* = 8)	2,8 (*n* = 26)
Ambulante Versorgung vor Ort (kein Transport)	60,2 (*n* = 1389)	63,1 (*n* = 291)	58,1 (*n* = 525)	60,6 (*n* = 573)

Eine Vorstellung in der Notaufnahme wurde Pflegeheimbewohnern (*n* = 146; 29,4 %) seltener empfohlen als Pflegebedürftigen in häuslicher Versorgung (*n* = 348; 37,6 %) bzw. Nichtpflegebedürftigen (*n* = 332; 36,6 %). Eine Empfehlung zur Weiterbehandlung durch den Hausarzt erhielten Pflegeheimbewohner (*n* = 117; 23,6 %) ebenfalls deutlich seltener als Pflegebedürftige in häuslicher Versorgung (*n* = 339; 36,6 %) und Nichtpflegebedürftige (*n* = 435; 44,0 %; Tab. 2).

### Probleme mit Dauerkathetern im Fokus

Eine Dauerkatheterversorgung, welche überwiegend männliche Patienten betraf, erfolgte bei 365 Einsätzen (*n* = 302; 84,8 %). Ein Großteil wurde vor Ort versorgt (*n* = 307; 84,6 %) und als nicht dringlich (PZC 0) eingestuft (*n* = 292; 81,3 %) (Supplement 3). Etwa die Hälfte dieser Einsätze betraf Pflegeheimbewohner (*n* = 191; 52,3 %) und weitere 140 Fälle (38,4 %) Pflegebedürftige in häuslicher Versorgung. In immerhin 34 Einsätzen (9,3 %) wurde eine Dauerkatheterversorgung bei Nichtpflegedürftigen durchgeführt.

## Diskussion

Von den insgesamt 2410 G-NFS-Einsätzen bei Personen mit einem Alter von 65+ Jahren erfolgte bei knapp 60 % eine Versorgung von Patienten, die pflegerische Leistungen in häuslicher oder stationärer Umgebung erhalten. Unabhängig von Pflegebedarf und Versorgungsort konnte über die Hälfte der Fälle durch eine Versorgung der G‑NFS vor Ort verbleiben. Bei mehr als einem Drittel (*n* = 191; 38,5 %) der G‑NFS-Einsätze im Pflegeheim bzw. bei 140 (15,1 %) der Pflegebedürftigen in der Häuslichkeit wurde eine Dauerkatheterversorgung durchgeführt. Dies traf in 8 von 10 Fällen auf männliche Patienten zu.

### Gemeindenotfallsanitäter als Alternative und Brücke zum Hausarzt

Etwa 60 % der Einsätze bei Älteren betrafen Pflegeheimbewohner und Pflegebedürftige in häuslicher Versorgung. In mehr als drei Viertel aller Einsätze wurde die Maßnahme Beratung durchgeführt, insbesondere bei Einsätzen in der Häuslichkeit. Dies verdeutlicht einen hohen pflegerischen und primärversorgenden Bedarf bei Pflegebedürftigen, der durch G‑NFS abgedeckt wird. Die sinkende hausärztliche Versorgung und zunehmende Arbeitsbelastung [10, 17] sowie der steigende Anteil älterer Menschen in Deutschland führen zu einer steigenden Nachfrage nach Hausbesuchen dieser Patientenklientel [10]. Es zeigt sich aber, dass die Zahl der Hausbesuche von Hausärzten rückläufig ist [4]. Untersuchungen aus Deutschland weisen darauf hin, dass Hausärzte eine Unterscheidung der Hausbesuche nach Dringlichkeit vornehmen und bei dringlichen Hausbesuchen an den Rettungsdienst verweisen, statt diese selbst durchzuführen [23]. Während es für Pflegeheimbewohner regelmäßige und teils präventive Hausarztbesuche gibt, liegt die Frequenz der Hausbesuche für Pflegebedürftige in häuslicher Versorgung möglicherweise niedriger. Weitere Studien aus Deutschland veranschaulichen, dass Hausärzte die Möglichkeit der Delegation an nichtärztliche Heilberufe zur eigenen Entlastung positiv bewerten, diese Möglichkeit jedoch wenig in Anspruch nehmen [2, 23]. Hier könnten Community-Health-Nurse(CHN)-Konzepte durch Interventionen wie Hausbesuche die Versorgungssituation verbessern [15]. Die Übernahme erweiterter pflegerisch-klinischer Aufgaben wie Untersuchungen und Diagnosestellungen [3] durch CHN könnten hier eine synergetische Versorgung darstellen und der vermehrten Inanspruchnahme des Rettungsdiensts durch pflegebedürftige Patienten entgegenwirken.

### Katheterversorgung bei Pflegeheimbewohnern und pflegebedürftigen Patienten in häuslicher Versorgung

Bei 153 (15,1 %) aller Einsätze wurde die Versorgung von Dauerkathetern aufgeführt, davon waren mehr als 90 % der Patienten pflegebedürftig und größtenteils männlich. Ein Transport der Patienten in weiterführende Versorgungseinrichtungen konnte bei acht von 10 Einsätzen (*n* = 307; 84,6 %) durch eine ambulante Versorgung verhindert werden. Eine Studie aus Bremen und Niedersachsen zeigt, dass 13,4 % der Pflegeheimbewohner mit einem Dauerkatheter versorgt sind [19]. Zu ambulant Pflegebedürftigen liegen unseres Wissens keine Zahlen vor. Es wird jedoch deutlich, dass Probleme mit einem Dauerkatheter ein zentrales Versorgungsproblem bei Pflegebedürftigen darstellen und dazu nicht selten Transporte in eine Notaufnahme erfolgen [9]. Während die Anlage eines Dauerkatheters oder die Versorgung von Dauerkathetern (regelmäßiger Wechsel) bei weiblichen Bewohnern in Pflegeheimen häufig durch das Pflegepersonal vor Ort erbracht wird, ist eine Dauerkatheterversorgung männlicher Bewohner meist mit einer Vorstellung in einer urologischen Praxis verbunden. Die Versorgung von Dauerkathetern kann durch Ärzte an examiniertes Pflegepersonal mit einer dreijährigen Ausbildung delegiert und somit ambulant durchgeführt werden. Vor dem Hintergrund des Fachpersonalmangels und der finanziellen Belastung von Pflegeheimen nimmt der Anteil des qualifizierten Pflegepersonals ab [23] und damit die Möglichkeit und Bereitschaft, Verantwortung für die Patienten zu übernehmen [18]. Diese personelle Situation veranschaulicht, dass eine Versorgungslücke für die betroffenen Patienten vorliegt. Der Einsatz der G‑NFS kann diese Versorgunglücke zwar schließen, es stellt sich jedoch die Frage, ob dies nicht eigentlich primär ärztliche und pflegerische Aufgaben wären, die u. a. von CHN durchgeführt werden könnten, um den Rettungsdienst für die Wahrung seiner originären Aufgabe zu entlasten.

### Transport

Die Nachforderung eines Rettungsmittels zum Transport in weiterführende Versorgungseinrichtungen konnte in 60 % aller Einsätze (*n* = 1389) vermieden werden. Damit wirkt der Einsatz der G‑NFS den zunehmenden Rettungsdiensteinsätzen [22] entgegen und führt zur Entlastung der Rettungswageneinsätze und folglich der Notaufnahmen [20]. Diese Entwicklung ist vor dem Hintergrund der stetig steigenden Anzahl geriatrischer Patienten in den Notaufnahmen [7, 11] von erheblicher Bedeutung und birgt sicherlich Vorteile für die Patienten: Diese können in ihrem gewohnten Umfeld verbleiben und erfahren eine Reduktion der psychischen Belastung durch die Vermeidung eines Aufenthalts in einer Notaufnahme und einer ggf. stationären Versorgung.

### Stärken und Schwächen

Stärke der vorliegenden Studie ist die Auswertung von insgesamt 2410 G‑NFS-Protokollen, die erstmals eine Unterteilung der Gesamteinsätze nach den unterschiedlichen pflegerischen Versorgungssettings ermöglichen. Als Limitation muss berücksichtigt werden, dass möglicherweise nicht zu allen Einsätzen ein G‑NFS-Protokoll ausgefüllt wurde. Der Abgleich verschiedener Daten im Rahmen der Qualitätssicherung lässt den Schluss zu, dass es sich aber eher um eine geringe Anzahl handelt. Die Einschätzung zur Versorgungssituation in der Häuslichkeit erfolgte subjektiv durch die G‑NFS und ist als Limitation zu betrachten, da die G‑NFS keine standardisierten Angaben zur Mobilität, vorliegender Erkrankungen, einer evtl. vorliegenden Pflegestufe oder Art und Umfang einer pflegerischen Versorgung durch einen Pflegedienst erfasst haben. Eine Aussage, ob es sich bei der Versorgung um ein akutes gesundheitliches Problem handelte oder ob die ergriffenen Maßnahmen aufgrund eines planbaren Ereignisses (z. B. Wechsel des Dauerkatheters) bei immer den gleichen Personen bzw. Einrichtungen erfolgten, lässt sich durch das Fehlen von Uhrzeit sowie Personen- und Adressbezug nicht ableiten. Ebenso lassen die Daten keinen Rückschluss auf erneute Alarmierungen des Rettungsdienstes durch die Patienten bzw. die Pflegenden nach der Versorgung durch die G‑NFS zu, wodurch möglicherweise die Transportquote unterschätzt wird. Da es sich beim G‑NFS um ein Modellprojekt handelt, kann nur die Versorgung einer begrenzten Region betrachtet werden.

### Schlussfolgerung

Erstmalig konnte mit dieser Studie die Versorgung von Pflegebedürftigen in häuslicher Umgebung durch G‑NFS dargestellt werden. In Regionen, die nicht über die zusätzliche Ressource G‑NFS verfügen, werden diese Patienten durch den „klassischen“ Rettungsdienst versorgt und aufgrund gesetzlicher Vorgaben auch häufig in eine Versorgungseinheit transportiert. Dies bindet Personal- und Zeitressourcen, folglich stehen die Rettungsmittel dann nicht für dringliche Notfälle zur Verfügung. Die Region dieses Modellprojekts umfasst sowohl städtische als auch ländliche Räume, folglich lassen sich die Ergebnisse vermutlich auch auf andere Regionen übertragen. Zu diskutieren ist, ob der Rettungsdienst für die Versorgung dieser niedrigprioritären und sektorenübergreifenden Einsätze zuständig ist und wie vor allem ältere Pflegebedürftige zukünftig bedarfsgerecht versorgt werden können.

## Fazit für die Praxis

- Ein hoher Anteil der Versorgung durch G‑NFS erfolgt bei älteren Patienten, mehr als die Hälfte ist pflegebedürftig.
- Häufig konnte ein G‑NFS-Einsatz einen Transport in eine nachfolgende Versorgungseinrichtung verhindern.
- Es besteht ein hoher pflegerischer und medizinischer Versorgungsbedarf ohne Vorliegen einer vital bedrohende Notfallsituation.
- Auf Basis der Ergebnisse ist davon auszugehen, dass auch bundesweit der Rettungsdienst zu einem nicht unerheblichen Anteil primärversorgende Aufgaben übernimmt.
- Grundsätzlich sollte diskutiert werden, wie neben der ärztlichen Profession weitere Heilberufe in die Akutversorgung eingebunden werden können, um auch in Zukunft die zunehmend größer werdende Gruppen Pflegebedürftiger bedarfsgerecht versorgen zu können.

### Supplementary Information






