# Supplementary material for: Frequency and characteristics of interventions by community paramedics on people in need of care: Analysis of 2,410 deployment protocols for people aged 65+ years
Source: Med Klin Intensivmed Notfmed. 2023 Dec 6;119(4):316–22. [Article in German] doi: 10.1007/s00063-023-01085-w (PMC11058764; doi:10.1007/s00063-023-01085-w)
Supplement: Supplementary file 1 [file 63_2023_1085_MOESM1_ESM.docx]

| **Demografische Daten** | |
| --- | --- |
| Geburtsjahr, Geschlecht | |
| **Sonstiges** | |
| First Responder Einsatz, Unterstützung RD, Patient aus Pflegeheim, häusliche Pflege, nicht pflegebedürftig | Einfachauswahl |
| **PZC (Patientenzuweisungscode)** | |
| PZC 0: kein Transport  PZC 1: sofortige intensivmedizinische Intervention  PZC 2: stationäre Aufnahme wahrscheinlich  PZC 3: ambulante Behandlung | Einfachauswahl |
| **Maßnahmen** | |
| Beratungsgespräch, Hilfe bei Selbstmedikation oder Inhalation, Wundversorgung, Kompressionsstrümpfe, Urinstix, Dauerkatheter anlegen/entfernen/spülen, Vitalparameter, i.v.-Zugang, Medikamentengabe, sonst. ärztliche Expertise hinzugezogen | Mehrfachauswahl |
| **Inanspruchnahme Telemedizin** | |
| Wurde genutzt: Entscheidung beeinflusst, erforderlich in Ermangelung (KV-Notdienst, Hausarzt), hat zur Nachforderung Rettungsmittel geführt  Wurde nicht genutzt: nicht erforderlich, kein Funkempfang, stand nicht zur Verfügung | Mehrfachauswahl |
| **Notwendigkeit eines Transports** | |
| Rettungswagen, Nachforderung NEF, Krankentransportwagen, Notfall-Krankentransportwagen, Begleitung eines Notfall-Transportes durch GNFS, Taxi/Mietwagen, Rollstuhltransport, privater Transport oder auf Verzicht auf Transport, Anforderung GNFS durch RTW/N-KTW/KTW | Einfachauswahl |
| **Empfehlungen** | |
| Vorstellung: Hausarzt, KV-Bereitschaftsdienst, niedergelassener Facharzt, Notaufnahme, Psychiatrie/psychiatr. Dienst, Information von Angehörigen, des ambulanten Pflegedienstes oder Krisenintervention | Mehrfachauswahl |
| **Kontaktaufnahme zum Hausarzt / KV-Bereitschaftsdienst durch Patienten** | |
| Hausarzt/KV-Bereitschaftsdienst wurde vom Pat. kontaktiert, Kontakt zum Hausarzt/KV-Bereitschaftsdienst nicht ausreichend:  Überlastung Hausarzt oder Überlastung KV-Bereitschaftsdienst  Patient an 112 verwiesen, Hausarzt/KV-Bereitschaftsdienst vom Pat. nicht erreichtKein Versuch vom Pat. Kontaktaufnahme Hausarzt/KV-Bereitschaftsdienst | Mehrfachauswahl |
| **Kontaktaufnahme zum Hausarzt KV-Bereitschaftsdienst durch G-NFS** | |
| Hausarzt/KV-Bereitschaftsdienst vom GNFS kontaktiert,  Hausarzt/KV-Bereitschaftsdienst vom GNFS nicht erreicht,  Kein Versuch vom GNFS der Kontaktaufnahme Hausarzt/KV-Bereitschaftsdienst | Mehrfachauswahl |
| **Einschätzung des GNFS zur Kategorisierung durch die Leitstelle** | |
| richtig, zu hoch oder zu niedrig kategorisiert, telefonische Fachberatung wäre ausreichend gewesen | Einfachauswahl |

Supplement 1: Angaben GNFS-Protokoll
