# Supplementary material for: Frequency and characteristics of interventions by community paramedics on people in need of care: Analysis of 2,410 deployment protocols for people aged 65+ years
Source: Med Klin Intensivmed Notfmed. 2023 Dec 6;119(4):316–22. [Article in German] doi: 10.1007/s00063-023-01085-w (PMC11058764; doi:10.1007/s00063-023-01085-w)
Supplement: Supplementary file 2 [file 63_2023_1085_MOESM2_ESM.docx]

| Supplement 2: Maßnahmen bei Einsätzen von Gemeindenotfallsanitätern, gesamt und stratifiziert nach Einsatzort | | | | | |
| --- | --- | --- | --- | --- | --- |
|  | Beratung | Hilfe bei Selbst-medikation | Medikations-gabe* | Versorgung Dauerkatheter | Wundver-sorgung |
| Altersgruppen | Gesamt (n=2.410) | | | | |
| 65-74 Jahre | 77,2% (n=474) | 16,8%  (n=103) | 20,7%  (n=127) | 11,1%  (n=68) | 2,6%  (n=16) |
| 75-84 Jahre | 76,4%  (n=764) | 17,9%  (n=179) | 16,8%  (n=168) | 15,3%  (n=153) | 1,9%  (n=19) |
| 85+ Jahre | 74,0%  (n=589) | 15,6%  (n=124) | 18,0%  (n=143) | 18,1%  (n=144) | 2,8%  (n=22) |
| Geschlecht |  | | | | |
| Männer | 70,8%  (n=843) | 12,6%  (n=150) | 13,6%  (n=162) | 25,4%  (n=302) | 2,8%  (n=33) |
| Frauen | 81,4%  (n=957) | 21,3%  (n= 250) | 22,5%  (n=265) | 4,6%  (n=54) | 2,0%  (n=23) |
| Gesamt | 75,8%  (n=1827) | 16,8%  (n=406) | 18,2%  (n=438) | 15,1%  (n=365) | 2,4%  (n=57) |
| Altersgruppen | Pflegeheim (n=496) | | | | |
| 65-74 Jahre | 53,6%  (n=45) | 10,7%  (n=9) | 14,3%  (n=12) | 40,5%  (n=34) | 3,6%  (n=3) |
| 75-84 Jahre | 46,3%  (n=81) | 7,4%  (n=13) | 12,6%  (n=22) | 40,6%  (n=71) | 2,9%  (n=5) |
| 85+ Jahre | 48,1%  (n=114) | 10,1%  (n=24) | 16,0%  (n=38) | 36,3%  (n=86) | 5,5%  (n=13) |
| Geschlecht |  | | | | |
| Männer | 44,0%  (n=132) | 8,0%  (n=24) | 10,0%  (n=30) | 55,7%  (n=167) | 3,7%  (n=11) |
| Frauen | 57,4%  (n=105) | 12,0%  (n=22) | 22,4%  (n=41) | 9,3%  (n=17) | 5,5%  (n=10) |
| Gesamt | 48,4%  (n=240) | 9,3%  (n=46) | 14,5%  (n=72) | 38,5%  (n=191) | 4,2%  (n=21) |
| Altersgruppen | Häusliche Pflege (n=926) | | | | |
| 65-74 Jahre | 74,7%  (n=121) | 13,6%  (n=22) | 14,8%  (n=24) | 17,3%  (n=28) | 4,9%  (n=8) |
| 75-84 Jahre | 83,7%  (n=324) | 18,9%  (n=73) | 15,2%  (n=59) | 16,0%  (n=62) | 1,6%  (n=6) |
| 85+ Jahre | 83,6%  (n=315) | 19,6%  (n=74) | 17,0%  (n=64) | 13,3%  (n=50) | 1,6%  (n=6) |
| Geschlecht |  | | | | |
| Männer | 79,3%  (n=353) | 14,2%  (n=63) | 11,9%  (n=53) | 24,0%  (n=107 | 2,9%  (n=13) |
| Frauen | 84,9%  (n=395) | 22,6%  (n=105) | 19,6%  (n=91) | 6,7%  (n=31) | 1,3%  (n=6) |
| Gesamt | 82,1%  (n=760) | 18,3%  (n=169) | 15,9%  (n=147) | 15,1%  (n=140) | 2,2%  (n=20) |
| Altersgruppen | Nicht Pflegebedürftig (n=988) | | | | |
| 65-74 Jahre | 83,7%  (n=308) | 19,6%  (n=72) | 24,7%  (n=91) | 1,6%  (n=6) | 1,4%  (n=5) |
| 75-84 Jahre | 82,0%  (n=359) | 21,2%  (n=93) | 19,9%  (n=87) | 4,6%  (n=20) | 1,8%  (n=8) |
| 85+ Jahre | 87,9%  (n=160) | 14,3%  (n=26) | 22,5%  (n=41) | 4,4%  (n=8) | 1,6%  (n=3) |
| Geschlecht |  | | | | |
| Männer | 80,3%  (n=358) | 14,1%  (n=63) | 17,7%  (n=79) | 6,3%  (n=28) | 2,0%  (n=9) |
| Frauen | 86,6%  (n=457) | 23,3%  (n=123) | 25,2%  (n=133) | 1,1%  (n=6) | 1,3%  (n=7) |
| Gesamt | 83,7%  (n=827) | 19,3%  (n=191) | 22,2%  (n=219) | 3,4%  (n=34) | 1,6%  (n=16) |

*Medikamentengabe und/oder Art der Verabreichung (po, sc, in, iv, supp)
