# Supplementary material for: Frequency and characteristics of interventions by community paramedics on people in need of care: Analysis of 2,410 deployment protocols for people aged 65+ years
Source: Med Klin Intensivmed Notfmed. 2023 Dec 6;119(4):316–22. [Article in German] doi: 10.1007/s00063-023-01085-w (PMC11058764; doi:10.1007/s00063-023-01085-w)
Supplement: Supplementary file 3 [file 63_2023_1085_MOESM3_ESM.docx]

|  | Dauerkatheter  (n=365) | Kein Dauerkatheter  (n=2.045) |
| --- | --- | --- |
| Einsatzort |  |  |
| Pflegeheim | 52,3% (n=191) | 14,9% (n=305) |
| Häusliche Pflege  Nicht-Pflegebedürftig | 38,4% (n=140)  9,3% (n=34) | 38,4% (n=786)  46,7% (n=954) |
| Alter in Jahren, Mittelwert (SD) | 82,0 (7,9) | 80,6 (8,2) |
| Geschlecht | (n=356) | (n=2011) |
| Männer | 84,8% (n=302) | 44,2% (n=889) |
| Frauen | 15,2% (n=54) | 55,8% (n=1122) |
| Dringlichkeit des Einsatzes | (n=359) | (n=1926) |
| PZC 0 | 81,3% (n=292) | 55,1% (n=1062) |
| PZC 1 | 1,1% (n=4) | 5,2% (n=101) |
| PZC 2 | 1,7% (n=6) | 24,4% (n=470) |
| PZC 3 | 15,9% (n=57) | 15,2% (n=293) |
| Einsatzdauer in Minuten, Mittelwert (SD) | 38,9 (16,9) | 47,0 (21,4) |
| Konsultation Hausarzt/ KV-Notdienst | 1,4% (n=5) | 7,3% (n=150) |
| Empfehlung weiterer Versorgung |  |  |
| Vorstellung in Notaufnahme | 10,4% (n=38) | 38,5% (n=788) |
| Vorstellung beim Hausarzt  Vorstellung beim Facharzt | 16,2% (n=59)  37,8% (n=138) | 40,7% (n=832)  2,2% (n=45) |
| Vorstellung KV-Notdienst | 0,5% (n=2) | 4,8% (n=99) |
| Transport | (n=363) | (n=2045) |
| RTW  N-KTW  KTW  Privater Transport  Kein Transport | 0,8% (n=3)  6,1% (n=22)  7,7% (n=28)  0,6% (n=2)  84,6% (n=307) | 11,9% (n=231)  12,2% (n=237)  14,0% (n=273)  5,4% (n=106)  55,6% (n=1082) |

Supplement 3: Charakteristika von Einsätzen in Abhängigkeit ob Versorgung eines

Dauerkatheters durchgeführt wurde
